# Analysis of Maternal Mortality in a Small Teaching Hospital Attached to Tertiary Care Hospital (A 10 yr review)

**DOI:** 10.4103/0970-0218.43234

**Published:** 2008-10

**Authors:** Rohul Jabeen Shah, Imtiaz Ali, Altaf Banday, Anjum Fazili, Imran Khan

**Affiliations:** Department of Community Medicine, S.K. Institute of Medical Sciences, Srinagar, India

## Introduction

Maternal death, as defined by the 9^th^ and 10^th^ revisions of the International Statistical Classification of Diseases and Related Health Problems (ICD), is “the death of a woman while pregnant or within 42 days of the end of the pregnancy, irrespective of the duration and site of pregnancy, from any cause related to or aggravated by the pregnancy or its management, but not from accidental or incidental causes.”(1)

The high level of maternal mortality in the developing world is an issue of public health concern. India accounts for over 20% of the world's maternal deaths(1) (SRS, GOI 1998). Maternal mortality is ascribed usually to complications that generally occur during or around labor and cannot be accurately predicted. The major causes of maternal mortality are hemorrhage, sepsis, hypertensive disorders, obstructed labor, and abortions. All of these causes are mostly preventable through proper understanding, diagnosis, and management of labor complications.(2)

Although varying levels of maternal mortality are reported in hospital settings, which vary from one state to another, we have no such data available from our valley. This study is an attempt to collect such information.

## Materials and Methods

This retrospective study has been conducted by analyzing all hospital case records from the Obstetric and Gynecology Department at S.K. Institute of Medical Sciences (SKIMS) in Srinagar, a tertiary care hospital, from January 1997 to December 2006 for all pregnancy-related deaths. This hospital is a very small hospital with 24 functional beds in the ward and 4 beds in the labor room. The patients' demographic record including age, parity, birth interval, antenatal care record, type of delivery, type of previous deliveries, status of patient i.e., booked or not booked, condition in which the patient was admitted to the hospital, distance from hospital, process chart during hospital stay, interventions, problems encountered, and probable cause of death as assessed by reviewing case sheet records and final death certificates were recorded. No autopsy is performed routinely at death in the hospital due to socio-religious reasons and hence autopsy reports to confirm the cause of death were not available for such cases. Necessary permissions and clearances to access the hospital records were obtained from the Medical Superintendent of the hospital. Odds ratio, confidence interval, and tests of significance were applied by using the statistical software package STAT 4U.

## Results

A total of 28,094 females were admitted between January 1997 and December 2006, of which 22,094 females delivered and 1,712 had abortions. The rest of the women were admitted for other reasons like pregnancy-induced hypertension, severe anemia, or for other obstetric reasons and were discharged after being treated. Of those who delivered in the hospital, 14,561 (65.90%) of the deliveries were by normal vaginal delivery and 7,533 (34.09%) were by lower segment cesarean section (LSCS). Ten maternal deaths were recorded during this period making the maternal mortality ratio around 47/100,000 live births. The major cause of death was hemorrhage (60%) followed by thrombo-embolism, amniotic fluid embolism, and fulminant hepatitis. A total of 1712 patients (6.09%) were admitted for abortions; 589 babies were stillborn making the still birth ratio 27.38/1000 live births. Those patients who were admitted for spontaneous or missed abortions underwent dilatation and curettage (D and C) according to the need.

Females in the age group of 30–35 years had a greater risk of dying than other age groups, a similar trend was seen in the age group above 35 years. The relationship is statistically significant (OR 0.086, 0.056 with C.I as 0.026–0.283 and 0.013–0.233, respectively for both age groups) as shown in Table 1.

**Table 1:** Maternal deaths in relation to age

Age in years	No. of deliveries (%)	No. of maternal deaths	OR*	CI*	RR*	CI*
15–20	2032 (9.45)	0	-	-	-	-
20–25	8718 (40.54)	0	-	-	-	-
25–30	9298 (43.24)	4	1.143	0.346–3.768	1	1–1.001
30–35	1161 (5.40)	4	0.086	0.026–0.283	0.997	0.994–0.999
> 35	296 (1.37)	2	0.056	0.013–0.233	0.994	0.983–0.999

* Values calculated for those age groups in which only maternal deaths occurred. CI = Confidence interval; OR = Odds ratio; RR = Relative risk

Deaths had no significant relation to the parity of the patient although primi gravidas had a greater risk of dying than 2^nd^, 3^rd^, or 4^th^ gravida with OR = 3.059 (95% C.I. = 0.735–12.732).

A majority (70%) of the deaths were recorded for patients who delivered by cesarean section (LSCS) as compared with only 30% of patients who delivered by normal vaginal delivery. Hemorrhage was the major cause of death (60%) followed by thromboembolism (20%), amniotic fluid embolism (10%), and fulminant hepatitis (10%). In all the patients, it was post-partum hemorrhage, except in one female who had central placenta previa and also had severe ante-partum hemorrhage, followed by post-partum hemorrhage (PPH). About 70% of the deaths occurred in patients who lived within a distance of 0–5 km and 90% of them were booked with the hospital. Maternal deaths over the years remained static i.e., one per year except in 2004 and 2006 when it was two per year. No deaths were recorded in the year 2003. LSCS rates over the years increased from 29.67% in 1997 to 36.07% in 2006. Conversely, the rate of normal deliveries decreased from 70.33% to 63.95% in 2006.

## Discussion

Pregnancy and labor, if not kept under constant vigil, can end in serious complications or even death at any moment. The death is a tragedy and carries with it a huge burden of grief and pain for the family, especially for the young ones the mother leaves behind. Half a million women die each year due to pregnancy-related complications and 95% of them come from developing countries.(2) Avoidance of unwanted births, proper antenatal care by trained staff supported by institutional quality care, and delivery coupled with the empowerment of women has made maternal deaths during pregnancy a rare phenomenon in the industrialized world. In the developing world, however, it is still a commonly encountered phenomenon, where even these figures are considered as underestimates because of under reporting from various countries. There is no systematic mechanism of death reporting in general and particularly of maternal deaths in our country, especially in rural areas and thus it becomes extremely difficult to assess the maternal morality rate.

The maternal mortality ratio in our study was 47/100,000 live births, which is much lower compared with other studies.(1,2) Our results are more or less comparable to rates from tertiary care hospitals in South Asian countries or developed countries. Berg, *et al.* in the United States reported the maternal mortality rate as 10.3 in 1991 and 12.9 in 1997 per 100,000 live births.(3) In a study conducted in a tertiary care hospital in a neighboring country, Pakistan, Begum, *et al.* showed the maternal morality rate as 12.7/1000 live births.(2) The maternal mortality rate from a study conducted in Ethiopia was 9.6/1000 live births. The reason for the low maternal morality rate in this study is that our hospital entertains regular, booked cases only and provides good antenatal care with backup investigative facilities for early diagnosis of any risk factor and consultancy for its management is available for high-risk pregnancies. Rarely, un-booked patients from surrounding areas and from peripheral hospitals are accepted except under emergency conditions if they are referred or brought in for delivery.

In our study, a higher number of deaths were recorded in the 25–30 year age group and with an odds ratio (OR) of 1.143. We also found that primi gravidas have a greater risk of dying (OR 3.059) than multigravidas. In her 10-year study from a medical college hospital, Vimal showed 54.9% deaths in the age group of 20–30 year olds, 29% deaths in primigravidas, and approximately 56% deaths among multigravidas.(4) Begum's study reports that 61.5% of maternal deaths occurred in the age group >30 years old and 69% of maternal deaths occurred in grand multiparas; these women lived at a distance of 10–100 km from the hospital. In our study, most of the women lived within a radius of 0–5 km and 90% of them were booked cases. More deaths occurring in this radius is attributed to the fact that more than half of the women lived within a 0–5 km range and sought admission for delivering in this hospital. In a rural district setting, Font, *et al.* showed a lifetime risk between the age groups as relatively stable and declining with no higher risk of mortality with older age, although lower age group (15–19 years) values were higher than the rest(5) with a very wide confidence interval.

Data from hospital records revealed obstetric hemorrhage and sepsis as the leading causes of death. A study conducted in Safdarjang Hospital in New Delhi revealed that 120 maternal deaths occurred during the year beginning 1 July 2003 to 30 June 2004 with post-partum hemorrhage (26%) as the leading direct cause of death; 89% of cases were un-booked.(1) In our setting, obstetric hemorrhage was also the leading cause of death followed by other causes. Although sub total hysterectomies were performed on 4 patients to control post partum hemorrhage (PPH), and almost all those containing severe PPH received blood, they could not be saved despite medical and surgical interventions. In a similar study from a tertiary care hospital, the leading cause of death was obstetric hemorrhage followed by other causes;(2) up to 32.24% were reported from a medical college hospital in a 10-year study.(4) The study also revealed that the rate of LSCS over the years has increased to 36.07%. The reason may be that the consultants and resident doctors on duty are made more accountable for any still birth or maternal death. Socioeconomic status and educational status could not be ascertained from the case records to conclude that they have an impact on the maternal mortality rate.

Although hospital-based maternal mortality figures do not give the true picture in the community, they tend to provide a more thorough assessment of the underlying cause of death and contributing factors that provide useful data in planning various strategies or interventions. It is concluded that good quality antenatal care supported by assisted deliveries can reduce the maternal mortality rate. Timely identification and intervention of risk factors through proper referral to better health facilities will go a long way in the reduction of maternal mortality rates.
